# Consequences of mating with siblings and nonsiblings on the reproductive success in a leaf beetle

**DOI:** 10.1002/ece3.2103

**Published:** 2016-04-06

**Authors:** Thorben Müller, Caroline Müller

**Affiliations:** ^1^ Department of Chemical EcologyBielefeld UniversityUniversitätsstr. 2533615BielefeldGermany

**Keywords:** Cuticular hydrocarbon profiles, family‐specific variability, inbreeding depression, *Phaedon cochleariae*, polyandry

## Abstract

Choosing a suitable mating partner is crucial for the fitness of an individual, whereby mating with siblings often results in inbreeding depression. We studied consequences of mating with siblings versus nonsiblings in the mustard leaf beetle, *Phaedon cochleariae* (Coleoptera: Chrysomelidae), on lifetime reproductive traits. Furthermore, we analyzed whether cuticular hydrocarbon (CHC) profiles are family specific and could potentially influence the mating behavior of young adults. We hypothesized a reduced reproductive success of females mated with siblings and a more rapid mating of males with nonsiblings. The hatching rate from eggs of sibling pairs was lower compared to that of nonsibling pairs, pointing to inbreeding depression. Furthermore, the number of eggs laid by females decreased over time in both sibling and nonsibling pairs. Interestingly, the CHC profiles and the body mass differed between families. However, the beetles did not avoid siblings and accepted them as readily as nonsiblings for mating in no‐choice tests. In summary, although it had negative consequences to mate a sibling and although siblings could potentially be recognized by their CHC profiles, the beetles did not show a delayed mating with siblings. Our results indicate that *P. cochleariae* beetles have not developed a precopulatory mechanism to avoid inbreeding, at least under the test conditions applied here. We predict that instead a polyandrous mating system and/or postcopulatory mechanisms might have evolved in this species by which inbreeding costs can be reduced.

## Introduction

To mate a suitable partner at a given time is an essential task in nature. Mate choice can have decisive consequences on the fitness of individuals and hence on the evolution of sexual selection in a population (Jennions and Petrie 1997; Kempenaers 2007). For many species, it is favorable to mate an unrelated partner to prevent inbreeding (Pusey and Wolf 1996; Keller and Waller 2002), as inbreeding can negatively affect various reproductive traits of the parent generation (Saccheri et al. 1996; Lihoreau et al. 2007; Vega‐Trejo et al. 2015) as well as the fitness of the offspring (Saccheri et al. 1998). Moreover, mating with siblings can result in a decreased heterozygosity and an increased extinction rate of small populations (Saccheri et al. 1998). However, the consequences of inbreeding are species‐specific and not necessarily negative (Kuriwada et al. 2011). Rather, costs and benefits are influenced by the ecological context of a species or population (Liu et al. 2014).

Also in insects, various species prefer unrelated mating partners to avoid costs of inbreeding depression (e.g., the cricket *Teleogryllus oceanicus* and the beetle *Colaphellus bowringi*) (Thomas and Simmons 2011; Liu et al. 2014). The mate recognition and preferences can be mediated by different chemical cues, including cuticular hydrocarbons (CHC) on the insect surface (Geiselhardt et al. 2009; Lihoreau and Rivault 2009; Thomas and Simmons 2011) or other contact pheromones (Simmons 1990; Herzner et al. 2006). For example, CHC profiles of the chrysomelids *Phaedon cochleariae* and *Chrysochus* spp. have been shown to be involved in male mate choice and to lead to assortative mating within a species or hybridizing species (Peterson et al. 2007; Geiselhardt et al. 2012). Apart from precopulatory inbreeding avoidance, alternative strategies to prevent costs of inbreeding are female polyandry (Arnqvist and Nilsson 2000; Tregenza and Wedell 2002) and postcopulatory mechanisms (Edvardsson et al. 2008; Bretman et al. 2009).

Relatively little is known about the development of the reproductive success over the adult lifetime of females that mated either closely related or unrelated males. For example, in the chrysomelid *Callosobruchus maculatus,* negative effects of inbreeding increase over time with the age of the females (Fox and Reed 2010). Females mated with a sibling might reduce their early investment in offspring and thus reserve more resources for the offspring of potential nonsibling males later in life (Bilde et al. 2007). Likewise, males should partition their sperm investment if it is limited (Wedell et al. 2002). However, it remains unclear which reproductive traits are mainly affected by inbreeding and when negative effects of inbreeding become evident during life.

Besides the relatedness of the parents, genetic and phenotypic differences between families or populations may determine the extent of inbreeding depression, the mate choice behavior, differences in chemical compounds which can mediate kin recognition, and the reproductive success in general (Fox and Scheibly 2006; Herzner et al. 2006; Lihoreau and Rivault 2009). For example, the sex pheromone composition of European beewolves is more similar within than among families, pointing to a heritable component (Herzner et al. 2006). Population‐dependent differences in inbreeding depression could be detected in the seed beetle *Stator limbatus* (Fox and Scheibly 2006). In certain families or populations, inbreeding depression may occur earlier than in others, which can have consequences on the next generation.

**Figure 1:**
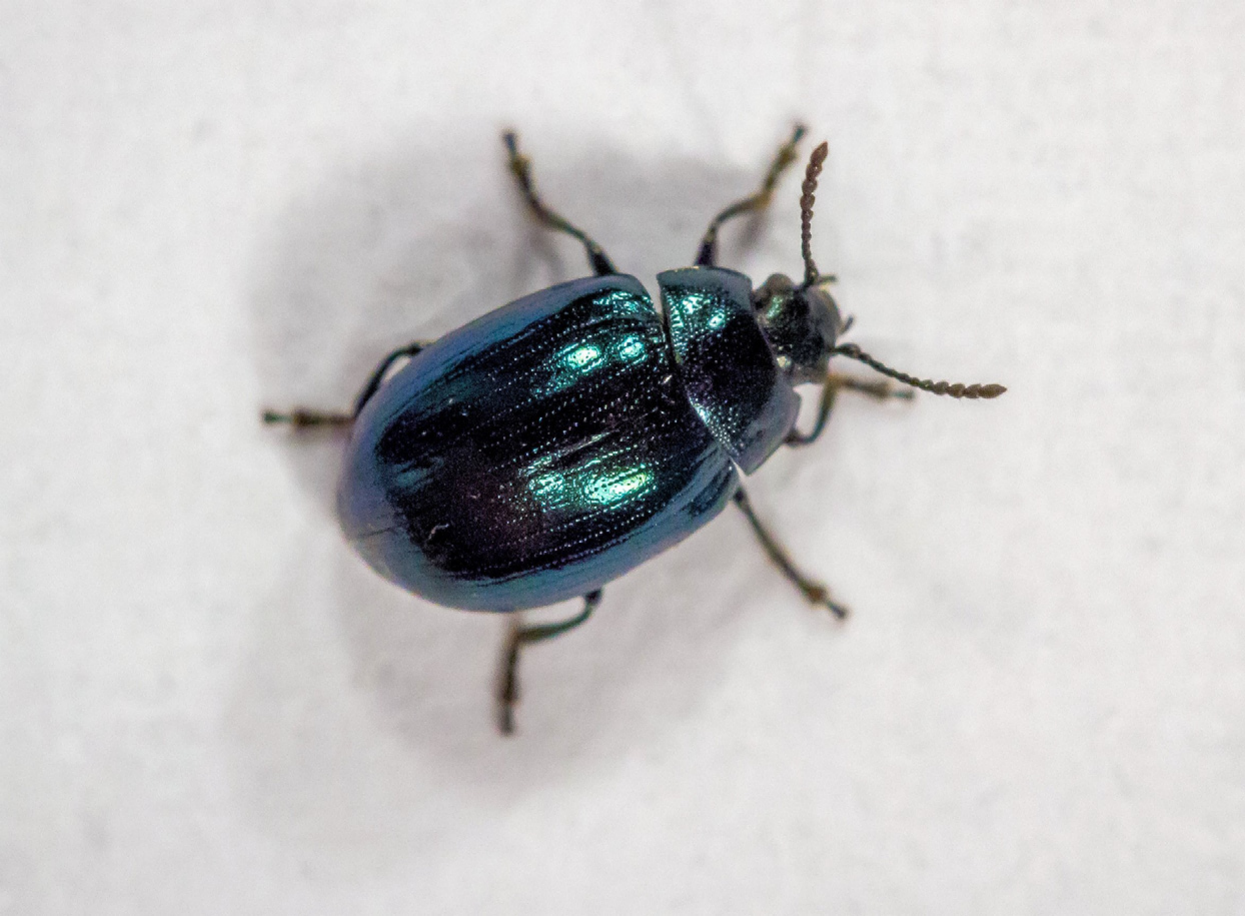
*Phaedon cochleariae*, the mustard leaf beetle (Coleoptera: Chrysomelidae), can reach a body length of 3–4.5 mm and is a herbivorous specialist for Brassicaceae, on which it can reach a pest status. This photograph of an adult female was taken by T. Müller, F. Bien, and C. Engelbrecht.

We aimed to investigate the consequences of mating with siblings versus nonsiblings on lifetime reproduction and mating behavior. Therefore, we used *P. cochleariae* as study organism (Fig. 1), which occurs in nature on watercress batches at the shore of small streams in different population densities. Due to water movements and the low dispersal ability of the beetles, closely related individuals can potentially remain on a plant batch and are, therefore, prone to inbreeding. Furthermore, *P. cochleariae* males actively search for females and are likely the choosy sex (Geiselhardt et al. 2012; Otte et al. 2015), but apart from mating adults do not socially interact with conspecifics. To detect potential inbreeding depression in this species, we studied the consequences of mating with siblings versus nonsiblings on various growth and reproductive traits over adult lifetime. To test whether CHC profiles may serve as recognition cues to distinguish between sibling and nonsibling mating partners, we analyzed these profiles from males and females of different families. Furthermore, to study potential precopulatory mechanisms of inbreeding prevention, we investigated the mating latencies and acceptance of siblings versus nonsiblings. We hypothesized that mating with a sibling negatively affects one or more reproductive traits as found in other chrysomelids (Fox et al. 2007), that females of sibling pairs invest less in their offspring than nonsibling pairs (Bilde et al. 2007; Vega‐Trejo et al. 2015) and that the reproductive success decreases over lifetime. As *P. cochleariae* females frequently lay eggs when unmated, we also measured the egg mass of virgin beetles as indicator for maternal investment, expecting that virgin beetles should invest less in unfertilized eggs than mated females in fertilized eggs. Furthermore, we predicted family‐specific CHC profiles that might serve as recognition cues for males in the mate choice process. Finally, we assumed that beetles should accept a nonsibling partner more readily for mating than a sibling to avoid costs of inbreeding.

## Material and Methods

### Study organism

We collected individuals of *P. cochleariae* in different parts of Germany (south of Bielefeld at the Furlbach, at side arms of the river Main close to Randersacker, and in the Botanical Garden of Berlin‐Dahlem). Populations were mixed and reared for several generations in at least three ventilated plastic boxes (20 × 20 × 6.5 cm) in a climate cabinet (20°C, L16:D8, 65% r.h.) as rearing stock. Each year, we collected *P. cochleariae* in the field near Bielefeld and introduced them to the rearing stock to maintain genetic diversity. We kept about 200 beetles per box in a nearly balanced sex ratio, as found in the field, and provided them with leaves of *Brassica rapa* L. ssp. *pekinensis* var. Michihili (Brassicaceae) ad libitum. Plants were grown from seeds (Kiepenkerl; Bruno Nebelung GmbH, Konken, Germany) in pots (12 cm diameter) with composted soil in a greenhouse (L16:D8, 60% r.h.). Only leaves of nonflowering, 8–10‐week‐old plants were provided as diet.

### Experimental setup

We took pupae from three plastic boxes of the rearing stock and kept them separately in Petri dishes (5.5 cm diameter). These individuals served as experimental parent generation (F0) of the first study generation (F1). After eclosion of the adults, we reared five females and five males originating from different boxes as pairs to generate the five families (A–E) of the study generation. We provided the beetles with disks (2.5 cm diameter) of middle‐aged leaves of cabbage, which were kept moist in wet sponge rubber. The leaf disks were exchanged every to every other day. The females usually gnaw little holes in the leaf epidermis and lay single eggs in these cavities, which are covered with secretion (Müller and Rosenberger 2006). More than 20 eggs per day can be produced by females from day 10 onwards (Müller and Müller 2016). From day 20 of the adult lifetime onwards, we collected leaves with eggs daily for 8–10 days. These leaves were kept in Petri dishes (9.5 cm diameter) until hatching of the larvae (F1). We reared young larvae in groups of about 20 individuals per Petri dish (9.5 cm diameter), separated by family. From the third larval stage on, we kept them in similar group sizes in larger Petri dishes (14.5 cm). The pupae were separated and kept individually in small Petri dishes (5.5 cm diameter). After adult eclosion, cuticle hardening, and determination of the sex, we assigned the beetles of each of the five families to three groups for the following experiments. (1) To study the development of the body mass and reproductive traits in dependence of mating with a sibling versus nonsibling, we kept pairs of one female and one male belonging to one family (siblings) or to different families (nonsiblings) in Petri dishes (5.5 cm diameter) for their lifetime (in total, 12 sibling and 12 nonsibling pairs per family). The pairs mate multiple times throughout life. For comparison, we kept additionally isolated virgin females without a mating partner (six females per family). (2) To determine the CHC profiles of each family, we kept groups of five females or five males of each family, respectively, in three Petri dishes (9.5 cm) per family and sex (in total, 15 females and males per family). (3) For the mating experiment, we kept the beetles separately for about 20–24 days in Petri dishes (5.5 cm diameter) (24 females and 24 males per family).

### Measurements of development of adult body mass and reproductive success

To measure the development of various traits with adult aging, we determined the adult body mass as well as the number of laid eggs, the egg mass, and the hatching rate from eggs of sibling and nonsibling pairs as reproductive traits repeatedly over the adult lifetime. We measured each trait four times, starting 2 weeks (W2) after adult emergence and from that time on every 2 weeks up to week 8 of the adult lifetime (W4, W6, W8), which is the life expectancy of the majority of beetles under our laboratory conditions (Müller and Müller 2015). Every pair stayed together from day 2 or 3 after adult eclosion until death. We measured the body mass of each beetle to the nearest 0.1 mg. The body mass of insects is often crucial for the reproductive output (Smith and Fretwell 1974; Koch and Meunier 2014), and thus, we used it as additional reproductive and fitness‐related trait. Furthermore, we counted the number of eggs laid over four consecutive days (W2 day 14–17; W4 day 28–31; W6 day 42–45; W8 day 56–60). During this period, the leaf disk was daily exchanged after counting the eggs. To determine the average egg mass, we carefully separated 6–10 eggs per female from the host leaves and weighed them individually on a precision balance (ME36S; Sartorius AG, Göttingen, Germany). Therefore, eggs from six to eight females of the sibling and nonsibling pairs were measured per family. Additionally, we determined the egg mass of 8–10 eggs of six unmated virgin females per family once, when females were about 20 days old. Virgin females of *P. cochleariae* also lay eggs but do not place them on the leaf but instead on the filter paper or the Petri dish (T. Müller, pers. observ.). To determine the hatching rate from eggs laid by females kept in sibling and nonsibling pairs (six to eight per family), we counted the eggs laid on a leaf disk within one day by a given female, kept this leaf for about 1 week in an additional Petri dish (5.5 cm diameter) and counted the hatched larvae. After the study time of 8 weeks, about half of the beetles were still alive.

### Analysis of CHC profiles

To investigate whether CHC profiles differ between females and males of different families and could potentially serve as mate recognition cues, we extracted and chemically analyzed CHCs from individual adults. Therefore, we kept groups of five beetles per sex and family together in Petri dishes (9.5 cm diameter) for about 20–24 days, analogous to the age of beetles used in the mating experiment. Between seven and 11 individuals per sex and family were taken from all three rearing Petri dishes which existed per family and sex. We starved beetles for about 7 h, before killing them in separate Eppendorf tubes by freezing them at −20°C. After thawing the beetles at room temperature for 15 min, we added 70 *μ*L dichloromethane to every beetle. As internal standard, we added 5 *μ*L *n*‐eicosane solution (0.1 mg/mL in hexane). After an incubation time of 10 min at room temperature, 55 *μ*L of each sample was transferred into a vial (0.2 mL; Fisher Scientific, Schwerte, Germany) and the CHCs were analyzed. The CHC profiles were measured by gas chromatography coupled with mass spectroscopy (Focus GC and DSQII MS, Thermo, 515 Electron Corporation, S.o.A. Rodano, Italy) on a VF‐5 ms column (30 m × 0.2 mm ID, 10 m guard column; Varian, Palo Alto, CA), using the temperature gradient as in Geiselhardt et al. (2009, 2012). We measured an alkane standard mix (C10‐C40; Sigma Aldrich, Karlsruhe, Germany) under the same conditions to calculate the retention indices of the compounds (Kovats 1958). Compounds were putatively identified by comparing their retention times and mass spectra to those published by Geiselhardt et al. (2009). Relative quantities of compounds were calculated per beetle.

### Mating experiment

Beetles for the mate choice experiment were separately reared for about 20–24 days, in accordance with previous mate choice experiments with this leaf beetle species (Geiselhardt et al. 2009, 2012; Otte et al. 2015). To determine whether beetles show a different latency until mating with siblings versus nonsiblings, we performed a no‐choice test where either a sibling or a nonsibling beetle was offered as potential mating partner. A no‐choice assay was chosen as in Geiselhardt et al. (2012) to reflect the natural situation in which a male meets one female and either mating occurs or not. Each beetle was only used once. We tested 12 sibling and 12 nonsibling pairings per family. For the 12 nonsibling pairings, we used three mating partners of each of the other four families. We conducted the mating tests in Petri dishes (5.5 cm diameter). The edges of the dishes were coated with Teflon (Whitford GmbH, Diez, Germany) and lined with filter paper, which was replaced after every test. We placed the Petri dishes in a light tent (60 cm × 60 cm; fitTek^®^, Hong Kong Special Administrative Region of the People's Republic of China) to avoid external disturbances. Three lamps (20 Watt) uniformly lightened the test setup. The order of mating setups in the mating experiment was randomized, as calculated by R (R Developmental Core Team, 2014) with the R package “randomizeBE” (Labes 2015). At the beginning of the test, we placed one male in the centre of the Petri dish. After an acclimatization time of about 2 min, a female was added. Subsequently, we measured the time until first mating and the total mating duration within the test time of 45 min. Additionally, we measured the body mass of each individual, as body mass and size can affect the mating behavior of several insect species (Arnqvist et al. 1996; Engqvist et al. 2014; Kanuch et al. 2015).

### Statistical analysis

**Figure 2:**
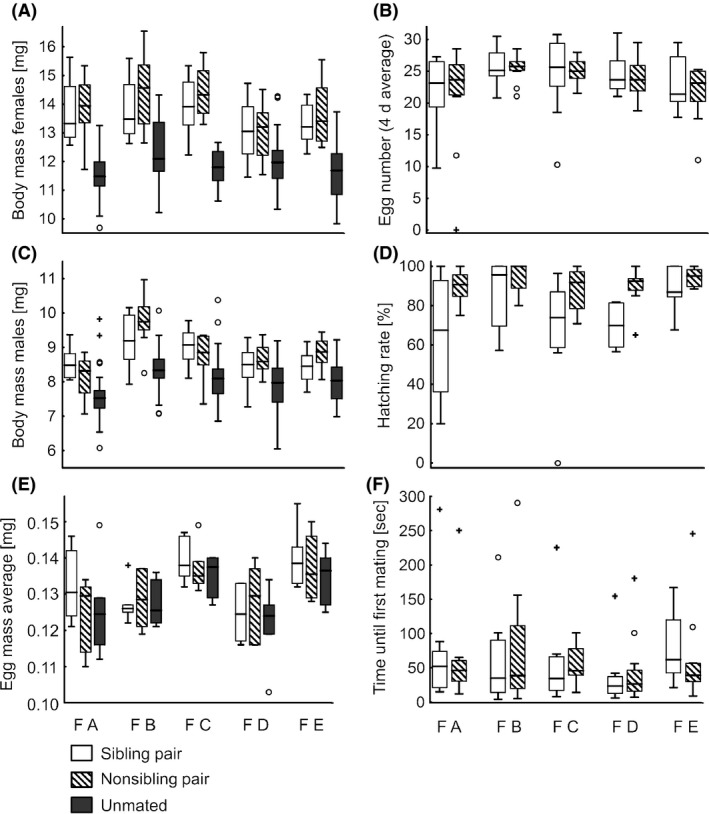
Impact of the family and the mating partner on A) female body mass (*n*_Sibling/Nonsibling_ = 11–12, *n*_Unmated_ = 24), C) male body mass (*n*_S/N_ = 11–12, *n*_U_ = 24), B) number of laid eggs per female, average over 4 days (*n *=* *12 for all families and pairs), E) average egg mass of six to 10 eggs per female (*n *=* *6 for all families, pairs and unmated individuals), D) the hatching rate (*n *=* *8 for all families and pairs), and F) the time until first mating (*n *=* *12 for all families and pairs) of about 2‐week‐old *Phaedon cochleariae* (for statistical analyses see Table 1). The boxes show the median (line) as well as the 25th and 75th percentiles. The whiskers expand to the minimum and maximum value without outliers; outliers are depicted as circles and extreme values as asterisks. F – Family (A–E).

**Figure 3:**
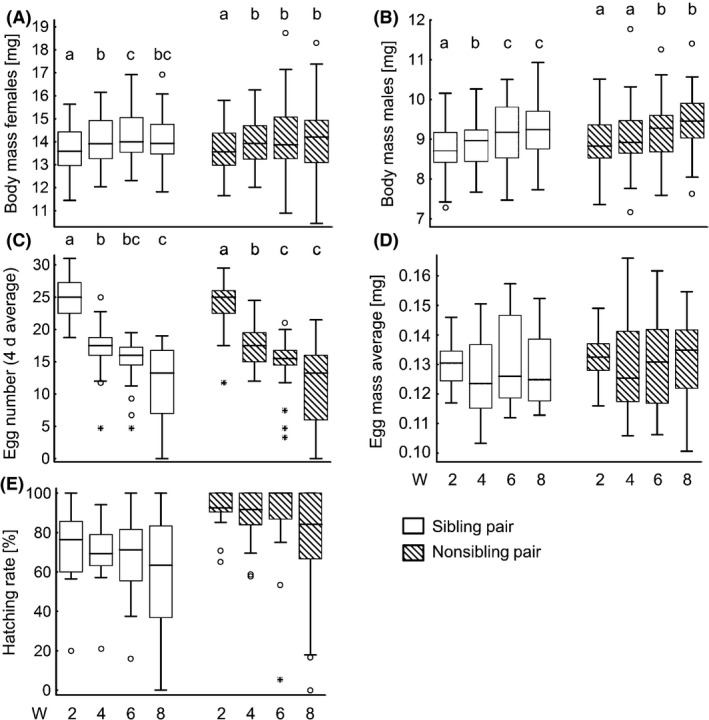
Development of the A) female body mass (*n*_S_ = 39, *n*_N_ = 43), B) male body mass (*n*_S_ = 39, *n*_N_ = 34), C) number of laid eggs per female, average over 4 days (*n*_S_ = 45, *n*_N_ = 45), D) average egg mass of six to 10 eggs per female (*n*_S_ = 16, *n*_N_ = 20), and E) hatching rate (*n*_S_ = 18, *n*_N_ = 25) of *Phaedon cochleariae* over 8 weeks, separately analyzed for sibling (*S*) and nonsibling (*N*) pairs (repeated measurement calculations). Significant differences (*P *<* *0.05) are indicated with different lower case letters. The boxes show the median (line) as well as the 25th and 75th percentiles. The whiskers expand to the minimum and maximum value without outliers; outliers are depicted as circles and extreme values as asterisks. W – Week.

**Figure 4:**
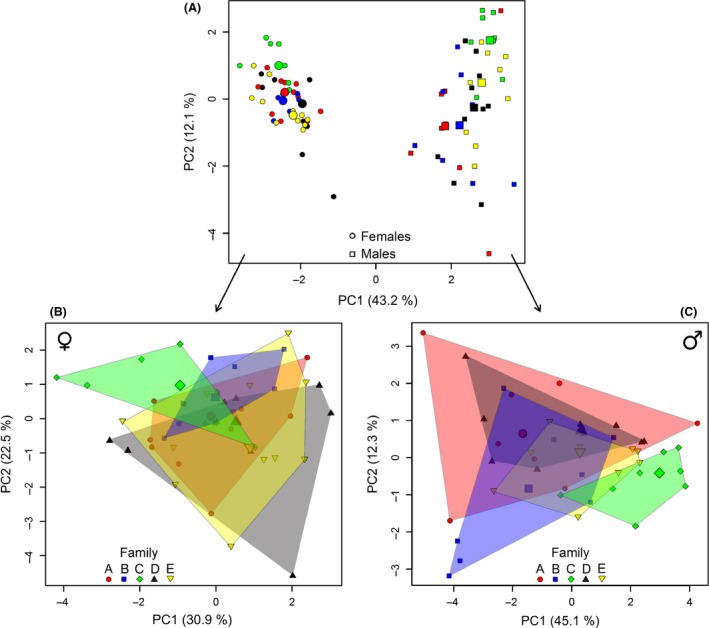
Cuticular hydrocarbon profiles shown as score plots of principle component analyses (PCA) of A) all individuals, B) females, and C) males of *Phaedon cochleariae*. The total variance explained by the PCs is shown as percent for each axis, and the score median of each group is depicted as larger symbol. Only compounds which were detectable in at least 50% of the individuals tested per family or sex were included in the PCA (15 compounds for all individuals, nine compounds for females, and 14 compounds for males).

**Figure 5:**
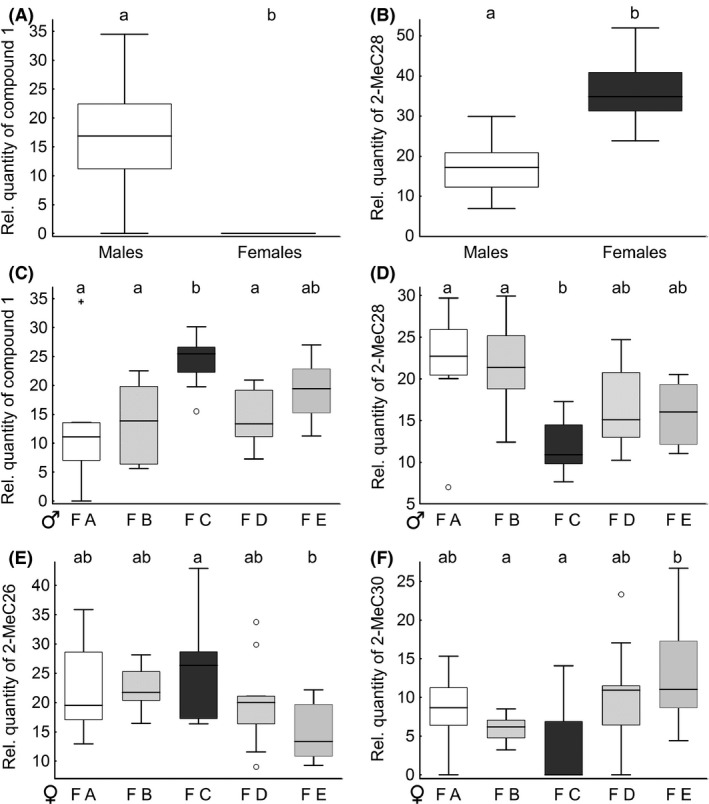
Relative quantities (in %) of selected cuticular hydrocarbons of *Phaedon cochleariae* of A) all females and B) males (*n*_Male_ = 44, *n*_Female_ = 47; Mann–Whitney *U*‐test, *P *<* *0.001 for both compounds) and of C–F) families (A–E) within males (C, D) and females (E, F) (*n *=* *7–11 per family and sex, tested with MANOVAs followed by univariate ANOVAs and pairwise comparisons, Bonferroni–Holm method, see Table 2). Significant differences (*P *<* *0.05) are indicated with different lower case letters. The boxes show the median (line) as well as the 25th and 75th percentiles. The whiskers expand to the minimum and maximum value without outliers; outliers are depicted as circles and extreme values as asterisks. Rel. – Relative, F – Family.

We performed statistical analyses with the statistical programs R (R Developmental Core Team, 2014) and SigmaPlot 11.0 (Systat Software, Erkrath, Germany). The datasets were tested for variance homogeneity and data distribution with Shapiro–Wilk or Levene's tests. The statistical program Statistica 10 (Statsoft, Hamburg, Germany) was used to generate Figures 2, 3, 4, 5.

To test whether family affiliations of females or males (A–E) as well as mating combinations (sibling or nonsibling pair), used as fixed factors, have an effect on the adult body mass, egg number, egg mass, or hatching rate at W2, we calculated generalized linear models (GLM). In separate GLMs, we used male and female adult body mass, egg number, egg mass, or hatching rate, respectively, as dependent variables and the mating partner (sibling pair, nonsibling pair, or unmated, the latter only for the body mass and the egg mass), the family of the females, and the interaction (mating partner × family) as fixed factors. For each model, we used the most appropriate error structure (Gaussian or gamma) and link function (identity, inverse, or log), depending on the respective data distribution. Analysis of deviance tables were calculated on the basis of the chosen error structure with either *F* (Gaussian) or *χ* ^2^ (gamma) statistics. The GLMs were conducted with R (R Developmental Core Team, 2014), using the R package “car” (Fox and Weisberg 2014).

To test how the body mass and reproductive traits of either sibling or nonsibling pairs develop over time (W2–W8) independent of family affiliation, we performed either a Friedman repeated‐measure ANOVA on ranks or an one‐way repeated‐measure ANOVA, depending on the respective data distribution, using SigmaPlot 11.0 (Systat Software). As family association was not the focus of this specific question, we pooled the data of all families to gain sufficient sample sizes until the end of the experiment (W8). The development of female and male body mass of sibling pairs and the egg mass of sibling and nonsibling pairs were analyzed with one‐way repeated‐measure ANOVAs, followed by pairwise multiple comparisons (Holm–Sidak method). The development of female and male body mass of nonsibling pairs, the egg number of sibling and nonsibling pairs as well as the hatching rate of sibling and nonsibling pairs were analyzed with Friedman repeated‐measure ANOVAs on ranks, followed by pairwise multiple comparisons (Tukey test).

To compare the CHC profiles of all individuals, we plotted a principal component analysis (PCA) after mean‐centering and scaling to unit variance of the data. Additionally, we performed PCAs for each sex separately. We included only compounds in the PCA, which were detectable in at least 50% of the individuals tested per family or sex. On the basis of a biplot (not shown), we identified the key compounds which mainly differed between the sexes or between the families within sex, respectively. Relative quantities of three compounds, which differed mostly between the sexes, were compared between females and males with Mann–Whitney *U*‐tests, because data were not normally distributed. For each sex separately, we calculated a multivariate analysis of variance (MANOVA) with the relative quantities of the three most decisive compounds (revealed from the biplot) as dependent variables and the family as factor. Subsequently, univariate analyses of variance (ANOVAs) were performed, followed by pairwise multiple comparisons (Bonferroni–Holm method), to specify the influence of the families on each compound. The PCAs, MANOVAs, and *U*‐tests were conducted with R (R Developmental Core Team, 2014).

In an additional GLM (performed with R), we investigated whether the family (A–E) and the mating partner (sibling or nonsibling) influence the time until first mating (mating experiment; as dependent variable). The body mass of males and females were integrated as covariates.

## Results

### Body mass at W2 in dependence of mating and family

The body mass of adult females and males at W2 was significantly influenced by the mating (sibling, nonsibling pair, or unmated). Unmated females were on average 13.4% and unmated males 8.8% lighter compared to mated conspecifics (Table 1, Fig. 2A and C). Moreover, the family affiliation of both sexes also determined their body mass, with individuals belonging to family B having on average the highest body mass, being 4.5% heavier in females and 7.3% in males compared to the other families. Additionally, the body mass of females was significantly impacted by the interaction of the family affiliation and the mating partner.

**Table 1:** Influence of the mating partner (sibling, nonsibling partner, and, where relevant, unmated), the family affiliation, and their interaction (fixed factors) on the dependent variables a) female body mass, b) male body mass, c) egg number, d) egg mass, and e) hatching rate of *Phaedon cochleariae*, two weeks after adult eclosion, calculated by generalized linear models. For each model, the analysis of deviance table is listed with *F* (Gaussian) or *χ* ^2^ (gamma) statistics, shown with the respective error structure and link function. Significant *P*‐values are indicated in bold. F – Female, M – Male, Resid. – Residuals

		NULL	Mating partner	Family F	Mating × Family F
(a) Body mass	Resid. df	237	235	231	223
Females	Resid. dev.	2.672	1.421	1.312	1.219
Gamma identity	*P* (*χ* ^2^)		<**0.001**	<**0.001**	**0.032**
		NULL	Mating	Family M	Mating × Family M
(b) Body mass	Resid. df	237	235	234	223
Males	Resid. dev.	158.158	122.525	99.378	92.911
Gaussian identity	*F*		42.762	13.890	1.940
	*P* (*F*)		<**0.001**	<**0.001**	0.055
		NULL	Mating	Family F	Mating × Family F
(c) Egg number	Resid. df	119	118	114	110
Gaussian identity	Resid. dev.	2395.1	2387.6	2129.5	2121.7
	*F*		0.384	3.345	0.101
	*P* (*F*)		0.537	**0.013**	0.982
(d) Egg mass	Resid. df	89	87	83	75
Gamma identity	Resid. dev.	0.504	0.491	0.347	0.332
	*P* (*χ* ^2^)		0.250	<**0.001**	0.912
(e) Hatching rate	Resid. df	79	78	74	70
Gaussian identity	Resid. dev.	29.771	24.597	21.305	20.133
	*F*		17.989	2.861	1.018
	*P* (*F*)		<**0.001**	**0.030**	0.404

### Reproductive success at W2 in dependence of mating partner and family

The egg number at W2 was significantly influenced by the family affiliation of the female (Table 1, Fig. 2B). Females of family B laid the highest number of eggs and females of family A the lowest number (15.3% less eggs than females of family B). Neither mating partner (sibling vs. nonsibling) nor the interaction between mating partner and female family had an effect on the egg number. Nearly all unmated females laid eggs around day 20.

The egg mass differed significantly between females belonging to different families (Table 1, Fig. 2E), with females of family C and E laying the heaviest eggs, being on average 7.5% heavier than the eggs laid by females of family A, B, or D. Neither mating partner (sibling, nonsibling pairs, or unmated) nor the interaction between mating partner and female family influenced the egg mass.

The hatching rate was significantly impacted by the mating situation of the beetles (Table 1, Fig. 2D). From eggs laid by nonsibling pairs, 17.6% more larvae emerged than from eggs laid by sibling pairs. Furthermore, the family affiliation also had a significant influence on the hatching rate. Eggs of family B and E had on average a 14.3% higher hatching rate compared to the ones of other families. No larvae hatched from eggs laid by unmated females.

### Development of body mass over adult lifetime

The body mass of adult females paired with a sibling male (one‐way repeated‐measure ANOVA; time, *F*_3,114_ = 14.730, *P* < 0.001) and of females paired with a nonsibling male (Friedman repeated‐measure ANOVA; time, *χ* ^2^ = 18.712, *P *<* *0.001; Fig. 3A) was lowest at week 2 of adult lifetime and increased over time (on average 4.2% higher at W6 and 3.5% at W8 compared to W2). Likewise, the body mass of adult males significantly increased on average by 5.8% over the period of 8 weeks (paired with sibling: one‐way repeated‐measure ANOVA; time, *F*_3,114_ = 20. 377, *P *<* *0.001; paired with nonsibling: Friedman repeated‐measure ANOVA; time, *χ* ^2^ = 38.506, *P *<* *0.001; Fig. 3B).

### Development of reproductive success over adult lifetime

The average number of laid eggs significantly decreased over time of females paired with sibling males (decrease of 53.8%; Friedman repeated‐measure ANOVA; time, *χ* ^2^ = 98.783, *P *<* *0.001) and nonsibling males (decrease of 52.8%; time, *χ* ^2^ = 100.924, *P *<* *0.001; Fig. 3C). In contrast, the average egg mass did not change over time, in neither females paired with siblings (one‐way repeated‐measure ANOVA; time, *F*_3,45_ = 1. 143, *P* = 0.342) nor females paired with nonsiblings (time, *F*_3,57_ = 0. 575, *P* = 0.634; Fig. 3D). Likewise, the hatching rate of eggs laid by females paired with siblings (Friedman repeated‐measure ANOVA; time, *χ* ^2^ = 4.307, *P* = 0.230) and females paired with nonsiblings was unaffected by time (time, *χ* ^2^ = 6.344, *P *=* *0.096; Fig. 3E).

### CHC profiles

We could detect 15 chemical compounds and putatively identified 11 of them primarily as methyl‐branched alkanes with chain lengths of hydrocarbons from C17 to C35, which were in accordance with retention indices mentioned in Geiselhardt et al. (2009) (Table S1). The CHC profiles differed between males and females (Fig. 4A). The difference was mainly based on two unknown compounds and the 2‐methylalkane 2‐MeC28. Both unknown compounds could only be detected in males (Shapiro–Wilk tests, *P *<* *0.001; Mann–Whitney *U*‐test, compound 1, *W *=* *23.500, *P *<* *0.001; compound 2, *W* = 47.000, *P *<* *0.001; Fig. 5A), whereas the relative quantity of 2‐MeC28 was higher in females (Shapiro–Wilk test, *P *=* *0.030; Mann–Whitney *U*‐test, compound 1, *W *=* *24.000, *P *<* *0.001; Fig. 5B). Additionally, the CHC profiles of males and females were family specific (Fig. 4B and C). In males, the relative quantities of two unknown compounds and 2‐MeC28 significantly differed between the families (Table 2, Fig. 5C and D). The two unknown compounds are likely also CHCs with at least 17 C‐atoms. The CHC profiles of females showed significant differences in the relative quantities of 2‐MeC26 and 2‐MeC30, respectively, between the families (Table 2, Fig. 5E and F).

**Table 2:** Statistical differences in relative quantities of selected cuticular hydrocarbons (CHCs) of adult *Phaedon cochleariae* in dependency on the family (A–E) analyzed for each sex separately. MANOVAs were used to detect the influence of the family (as factor) on three compounds (as dependent variable) of the CHC profile of males and females (*n *=* *7–11 per sex and family). Univariate ANOVAs were subsequently calculated to detect the impact of the family on each compound. Significant *P*‐values are indicated in bold

	df	Wilks *λ*	Approx. *F*	Num df	Den df	*P*‐value
(a) MANOVA						
Male family	4	0.482	2.597	12	98.184	**0.005**
Female family	4	0.545	2.281	12	106.120	**0.013**
	df	Sum sp	*F*‐value	*P*‐value	Resid. df	Resid. sum sq
(b) ANOVA						
Male family						
Unknown compound 1	4	872.890	5.215	**0.002**	39	1632.090
Unknown compound 2^1^	4	2965.1	4.437	**0.005**	39	6515.400
2‐MeC28	4	589.150	5.927	**<0.001**	39	969.150
Female family						
2‐MeC26	4	506.800	2.915	**0.032**	42	1825.300
2‐MeC28	4	369.520	2.204	0.085	42	1760.000
2‐MeC30^2^	4	505.430	4.644	**0.003**	42	1142.870

Resid., Residuals.

^1, 2^Shapiro–Wilk test: ^1^ *P *=* *0.027 and ^2^ *P *=* *0.031.

### Mating behavior

All individuals mated within the test time of the mating experiment. The time until first mating was affected neither by the mating partner (sibling or nonsibling) (Null model, residual df = 119, residual deviance = 111.251; factor mating partner: residual df = 118, residual deviance = 109.776, *P*(*χ* ^2^) = 0.261), nor by the family affiliation of the partner (factor family of the female: residual df = 114, residual deviance = 105.507, *P*(*χ* ^2^) = 0.453; factor family of the male: residual df = 110, residual deviance = 100.890, *P*(*χ* ^2^) = 0.412) or the body mass of females (factor body mass of the female: residual df = 109, residual deviance = 99.903, *P*(*χ* ^2^) = 0.357) (Fig. 2F). The body mass of males had a slightly significant influence on the latency until first mating (factor body mass of the male: residual df = 108, residual deviance = 94.337, *P*(*χ* ^2^) = 0.030), whereby lighter males started quicker with mating. Of 120 pairs, 112 mated until the end of the test time of 45 min once they had begun to mate, whereas only five sibling and three nonsibling pairs interrupted the mating process but continued it later on.

## Discussion

Mating with siblings resulted in a reduced hatching rate of the offspring, which indicates clear signs of inbreeding depression in this species. Egg numbers decreased over the lifetime, in both sibling and nonsibling pairs, whereas the egg mass and the hatching rate were stable throughout life. The CHC profiles differed between the tested families in males and females. However, males of *P. cochleariae* did not reject siblings but, in contrast, accepted them as readily as nonsiblings for mating in no‐choice situations. Obviously, inbreeding is not strictly prevented by precopulatory avoidance mechanisms although beetles are potentially able to distinguish between siblings and nonsiblings, based on family‐specific CHC profiles.

The reduced hatching rate and thus the lower reproductive success of sibling pairs compared to nonsiblings of *P. cochleariae* (Fig. 2D, Table 2) can likely be explained by inbreeding depression [i.e., genetic effects of a decreased heterozygosity (Pusey and Wolf 1996; Keller and Waller 2002)], which occurs in various insect species, including other chrysomelids (Saccheri et al. 1996; Fox et al. 2007; Edvardsson et al. 2008; Liu et al. 2014). Alternatively, the postmating maternal investment could have been affected, which influences similar traits as inbreeding depression, namely quality and quantity of the offspring (Vega‐Trejo et al. 2015). However, as the egg mass was not affected by the mating partner and, moreover, did not differ between fertilized and unfertilized eggs of *P. cochleariae*, influences of mating (partners) on maternal investment are less likely in this species. The similar egg mass of fertilized and unfertilized eggs is also surprising given that unmated females were significantly lighter than mated females. This indicates that egg mass may be, at least in a constant environment with identical food conditions, a rather fixed trait in *P. cochleariae*.

Interestingly, unmated *P. cochleariae* females show a different oviposition behavior compared to mated females; they do not place their eggs in the veins of leaves, such as mated females, but instead not at all on leaves. Thus, these unmated females do not invest in food provisioning of offspring and may only lay eggs due to a high oviposition pressure. Because differences in reproduction between females mated with siblings versus nonsiblings were only found in the hatching rate but neither in the egg number nor in the egg mass, our study highlights the need to measure as many reproductive traits as possible to fully cover the consequences of inbreeding on the reproductive success of a species.

Over adult development, the body mass of females and males increased in both sibling and nonsibling pairs in *P. cochleariae* (Fig. 3). In contrast, the number of laid eggs significantly decreased over time, whereas the egg mass and the hatching rate remained unchanged. Towards the end of their lifetime, individuals may primarily invest their resources to keep up the metabolic rate but reduce investment in offspring. The constant egg mass not only between females of different mating partners but also over time strengthens the idea that egg mass is a fixed trait, as long as the environment does not change. Likewise, egg quality in terms of hatching success seems to stay constant over time. Obviously, there is no time‐dependent reduced investment in eggs of *P. cochleariae* females, in contrast to reproductive traits of other species (Fox and Reed 2010). Furthermore, the development of all observed reproductive traits over time was rather similar between sibling and nonsibling pairs. However, the hatching rate of eggs laid by sibling pairs was constantly about 14–22% lower than in eggs of nonsibling pairs over the entire observation period. A reduced reproductive output in inbreeding lines may become even more obvious in subsequent generations (Bilde et al. 2007). Overall, lifelong observations of different reproductive traits have been rarely performed in insects but can offer interesting insight in lifetime investment strategies into reproduction.

In our chemical analysis of *P. cochleariae* individuals, we could confirm sex‐specific differences in CHC profiles described earlier in this beetle (Geiselhardt et al. 2012). Apart from these CHC differences between sexes, we also found differences in the CHC profiles within sexes between families and thus a high intraspecific variation (Figs. 4, 5). As we do not have genetic data of the five families we used for our mating experiments, we cannot determine to which degree the individuals were related. Nevertheless, the significant differences in CHC compounds between families point to genetic differences between the studied families and to a heritable component in the biosynthesis and modification of CHC profiles. Likewise, family‐specific, heritable CHC profiles were also found in crickets (*T. oceanicus*) and fruit flies (*Drosophila serrata*) (Hine et al. 2004; Thomas and Simmons 2008). Next to the CHC profiles, also the body mass of females and males as well as the reproductive traits egg number, egg mass, and hatching rate differed significantly between the five families tested in this study. A high intraspecific variability is the basis of natural selection and can also impact larger scale ecological dynamics (Bolnick et al. 2011).

In contrast to our expectation, males did not accept unrelated (nonsibling) females more readily than related siblings. Interestingly, under certain conditions, males of *P. cochleariae* show assortative mating, as they prefer females fed with the same host plant over females fed with another host, which differ in their CHC profiles (Geiselhardt et al. 2012). Thus, males are potentially able to discriminate between females, whereby CHC profiles, which are influenced by the diet, play an important role as recognition cues (Geiselhardt et al. 2012). However, diets differing in fatty acid composition caused only slight differences in CHC profiles of *P. cochleariae* beetles, which were not sufficient to lead to a discrimination of mating partners (Otte et al. 2015). Whether the mating behavior of *P. cochleariae* is affected by differences in the CHC profiles or not likely depends on the specific qualitative as well as quantitative modifications of the chemical composition (Otte et al. 2015).

Moreover, lighter males showed a shorter latency to mate than heavier males. It is unclear whether this is due to constraints of heavier males or adaptive for light males, which may be more successful in mating situations without competition. In a dual‐choice situation, in which related and unrelated mating partners are offered, mating may occur more readily with nonsiblings in *P. cochleariae*. Additionally, the mating experience with one partner may affect the choice and mating behavior in a subsequent mating situation, as known for other insect species (Dukas 2005; Harris and Moore 2005) and as predicted by a theoretical model (Engqvist and Reinhold 2006).

A lack of precopulatory inbreeding avoidance, as shown under the test conditions used here for *P. cochleariae*, has also been found in other invertebrate species (e.g., Ruch et al. 2009; Tan et al. 2012). Because males and females mate multiple times in *P. cochleariae*, if given the possibility, and thus they less likely always mate siblings, there may be no need for costly precopulatory inbreeding avoidance by kin recognition. Indeed, risks of inbreeding depression can be reduced when females are polyandrous (Tregenza and Wedell 2002). Females can profit from polyandry by gaining direct benefits during mating or indirect genetic benefits (Fedorka and Mousseau 2002). Alternatively, individuals may only recognize during or shortly after mating whether the partner is closely related or unrelated. Future mating experiments, in which *P. cochleariae* females are mated with at least two males of siblings or nonsiblings in different combinations, as carried out by Tregenza and Wedell (2002), may reveal whether postcopulatory avoidance mechanisms exist in this leaf beetle.

In summary, we demonstrate that mating with a sibling led to a reduced hatching rate of the offspring in all families. Inbreeding was not avoided prior to the copulation at least under our test conditions, even though family members could be potentially recognized by their CHC profiles. Thus, female polyandry and/or postcopulatory mechanisms may reduce inbreeding costs in *P. cochleariae*. Furthermore, we detected a high intraspecific variation in growth, chemical profiles, and reproductive traits in *P. cochleariae*. The lifelong reproduction measurements demonstrate that the egg number was the only reproductive trait which decreased over time, both in sibling and nonsibling pairs. Our study reveals a complex insight in the mating behavior and the consequences of mating with siblings versus nonsiblings for the lifetime reproductive success of a leaf beetle.

## Conflict of Interest

None declared.

## Supporting information


**Table S1.** Mean relative quantities (in %, ±SD) of cuticular hydrocarbons of adult female and male *Phaedon cochleariae* (*n*_Female_ = 47, *n*_Male_ = 44).
