# Supplementary material for: Consequences of mating with siblings and nonsiblings on the reproductive success in a leaf beetle
Source: Ecol Evol. 2016 Apr 6;6(10):3185–97. doi: 10.1002/ece3.2103 (PMC4829044; doi:10.1002/ece3.2103)
Supplement: Supplementary file 1 — Table S1. Mean relative quantities (in %, ±SD) of cuticular hydrocarbons of adult female and male Phaedon cochleariae (n Female = 47, n Male = 44). [file ECE3-6-3185-s001.docx]

**Table S1:** Mean relative quantities (in %, ± SD) of cuticular hydrocarbons of adult female and male *Phaedon cochleariae* (*n*_Female_ = 47, *n*_Male_ = 44). RI – Retention index.

| RI | Compound^a^ | Females | Males |
| --- | --- | --- | --- |
| 1671 | unknown compound 1 | 0 ± 0 | 16.79 ± 7.64 |
| 1676 | unknown compound 2 | 0 ± 0 | 28.84 ± 14.78 |
| 1942 | 7-MeC19 | 0 ± 0 | 1.14 ± 1.32 |
| 2268 | 6,9-C23diene | 0.05 ± 0.35 | 5.46 ± 2.94 |
| 2273 | 9-C23ene | 0.16 ± 0.70 | 5.14 ± 2.73 |
| 2532 | 11-/13-MeC25 | 5.54 ± 3.86 | 1.01 ± 1.15 |
| 2662 | 2-MeC26 | 20.37 ± 7.03 | 5.78 ± 2.74 |
| 2702 | 13-MeC27-2-ene / 15-MeC27-4-ene /  15-MeC27-9-ene | 2.11 ± 3.30 | 2.69 ± 1.80 |
| 2729 | 13-MeC27 | 7.65 ± 3.49 | 2.01 ± 1.60 |
| 2862 | 2-MeC28 | 36.21 ± 6.77 | 17.35 ± 5.99 |
| 3063 | 2-MeC30 | 8.92 ± 6.00 | 3.82 ± 2.23 |
| 3130 | 13-MeC31 | 1.22 ± 2.44 | 0.79 ± 1.08 |
| 3496 | 21-MeC35-10/12_ene / 23-MeC35-12/14-ene | 0.30 ± 1.16 | 4.87 ± 4.45 |
| 3696 | unknown compound 3 | 4.11 ± 6.03 | 3.87 ± 4.05 |
| 3892 | unknown compound 4 | 13.36 ± 11.10 | 0.51 ± 1.92 |

^a^ Compounds were putatively identified based on comparisons of RI with Geiselhardt *et al.* (2009).
